# Key sugar transporters drive development and pathogenicity in *Aspergillus flavus*

**DOI:** 10.3389/fcimb.2025.1661799

**Published:** 2025-09-08

**Authors:** Raheela Yasin, Sayed Usman, Qijian Qin, Xiufang Gong, Bin Wang, Linqi Wang, Cheng Jin, Wenxia Fang

**Affiliations:** ^1^ Institute of Biological Sciences and Technology, Guangxi Academy of Sciences, Nanning, Guangxi, China; ^2^ College of Life Science and Technology, Guangxi University, Nanning, Guangxi, China; ^3^ State Key Laboratory of Microbial Diversity and Innovative Utilization, Institute of Microbiology, Chinese Academy of Sciences, Beijing, China

**Keywords:** *Aspergillus flavus*, sugar transporters, sugar metabolism, cell wall, pathogenicity

## Abstract

*Aspergillus flavus* is a ubiquitous filamentous fungus that poses significant threats as both a causative agent of invasive aspergillosis and a major source of crop contamination due to production of aflatoxin B1 (AFB1). Sugars are essential for fungal metabolism, cell wall biosynthesis, and virulence, yet sugar transporters (STPs) in *A. flavus* remain largely uncharacterized. In this study, we systematically investigated three putative STP genes (*G4B84_001982*, *G4B84_005374*, and *G4B84_009351*) by comprehensive functional characterization of gene deletion mutants. Growth assays revealed that *G4B84_001982* and *G4B84_005374* mediate uptake of diverse sugar substrates, while *G4B84_009351* appeared to be non-essential under tested conditions. Heterologous expressions in the hexose transport-deficient *Saccharomyces cerevisiae* strain confirmed their sugar transporter activity. Phenotypic analysis revealed that the Δ*1982* and Δ*5374* mutants showed pleiotropic defects, including impaired growth, reduced sporulation, delayed germination, increased sensitivity to cell wall stressors, and completely abolished sclerotium formation. Pathogenicity assays demonstrated that the two mutants exhibited attenuated virulence in both plants (crop seeds) and animal (*Galleria mellonella*) infection model. Our findings highlight the essential of two STPs in *A. flavus* development, stress tolerance, and pathogenicity, offering insights into sugar-mediated pathogenicity in this economically and medically important fungus.

## Introduction

*Aspergillus flavus* is an opportunistic pathogen capable of causing infections in plants, humans, and animals (Pal et al., 2014). As ranked fifth among world’s ten most feared fungi (Hyde et al., 2018), it is second leading cause of both invasive and non-invasive pulmonary aspergillosis. Moreover, the larger diameter of *A. flavus* conidia favors superficial infections as well (Rudramurthy et al., 2019). Recent reports on outbreaks of COVID - 19 patients confirmed the presence of *A. flavus*, which highlighted its pathogenic potential causing co-infections alongside the virus (Thompson Iii et al., 2020). Another major concern with *A. flavus* is its ability to produce secondary metabolites, mainly AFB1, a toxic carcinogen, immunosuppressant, teratogen, and mutagen. AFB1 contaminates pre- and postharvest crops such as cereals, oilseeds, spices, and nuts (Jeyaramraja et al., 2018). Maize and peanut are staple food crops throughout the world with 1.2 billion and 45.65 million metric tons of production, respectively, in 2023 (FAOSTAT available: http://faostat.fao.org/), particularly significant in developing countries in Africa and Asia where aflatoxin contamination is most severe (Andrade and Caldas, 2015). Several Outbreaks of aflatoxicosis have been reported in these regions where environmental conditions such as high humidity and climate changes favor the growth of *A. flavus*, leading to human fatalities and threatening food safety and security (Farombi, 2006). Thus, effective intervention to curb pathogenesis and AFB1 contamination remains a pressing challenge.

Sensing and transporting external sugars enable fungal pathogens to regulate downstream metabolic processes, which are vital for host colonization, survival, and the initiation of infection (Lingner et al., 2011). Sugars also provide the carbon skeletons required for cell wall biosynthesis (Ruiz-Herrera et al., 2006), which comprises approximately 40% of the fungal cell’s volume and is essential for survival and defense against environmental stressors (Tada et al., 2013). Furthermore, studies have also reported that aflatoxin synthesis is influenced by the rate of sugar transport across the plasma membrane (Davis and Diener, 1968), where glucose and sucrose are preferred ones (Shantha and Murthy, 1981). Thus, sugars especially glucose serve as indispensable regulators of fungal metabolism, cell wall biosynthesis, and secondary metabolite production, underscoring their critical role in the progression of fungal infections.

To optimize sugar transport (STP) and coordinate the external environment with internal metabolism, the fungus must respond to extracellular sugar levels by regulating genes that encode mono- and oligosaccharide STPs (Lingner et al., 2011). In structural terms, about 99% of the STPs of filamentous fungi belong to the major facilitator superfamily (MFS) (Yin et al., 2006). The primary structure of MFS members typically consists of 400 – 600 amino acid residues, and they share a conserved three-dimensional architecture and functional properties (Yan, 2015). The canonical MFS fold is characterized by 12 transmembrane (TM) segments arranged into two 6-TM domains-N-terminal and C-terminal-connected by a long, flexible intracellular loop. This structural organization is a hallmark of all currently recognized MFS proteins. Hexose sugar transporter proteins (Hxt) belong to the sugar porter family within the MFS group (Reddy et al., 2012). The best-studied Hxts are found in *S. cerevisiae*, where they include seventeen hexose carriers (Hxt1 - 17p) as well as Gal2p, Snf3p, and Rgt2p. The extensive network of Hxts exhibits varying affinities for glucose and other substrates, with their expression tightly regulated by extracellular glucose concentrations (Horák, 2013). *S. cerevisiae* Snf3p (*Sc*Snf3p) and Rgt2p (*Sc*Rgt2p) function as glucose sensors and are distinguished by their long intracellular C-terminal tails, which are believed to play key roles in intracellular signal transduction (Kim et al., 2013).

MFS transporters in pathogenic fungi play essential roles in sugar transport and virulence. In *Colletotrichum lindemuthianum*, *MFS1* is specifically expressed during the necrotrophic phase and is crucial for sugar utilization in the host plant (Pereira et al., 2013). In *Botrytis cinerea*, *Bc*mfs1 protects against plant defense compounds during infection and antimicrobial agents during saprophytic growth (Hayashi et al., 2002). *Aa*MFS19 in *Alternaria alternata* is required for resistance to oxidative stress and fungicides, as well as full pathogenicity (Lin et al., 2018). In *Ustilago maydis*, the sucrose transporter *Um*SRT1 has a higher affinity than the host maize transporter *Zm*SUT1, enabling direct sucrose uptake from the apoplast and evasion of glucose-triggered defenses (Wittek et al., 2017). Hxt1 in *U. maydis* is the primary hexose importer for glucose, fructose, and mannose, and may also function as a glucose sensor during biotrophic development and smut disease progression (Schuler et al., 2015). In human pathogens, STPs also contribute to host adaptation. In *Candida albicans*, the transceptor Hgt4 senses simple sugars, regulates other glucose transporters, and is required for hyphal growth; its disruption leads to reduced virulence (Brown et al., 2006). *C. albicans* GlcNAc transporter has also been identified as a regulator of hyphal development (Alvarez and Konopka, 2007). Based on these findings involving STP in fungal pathogenicity, the focus of this study was to investigate whether STPs in *A. flavus* are essential for efficient host sugar acquisition to fuel growth, stress adaptation, and contribute to virulence by integrating environmental nutrient signals into molecular pathways governing fungal physiology and pathogenesis. By functionally characterizing key STPs, we aimed to establish their direct contribution to the pathophysiology and molecular adaptation of *A. flavus* during infection.

In this study, we identified and characterized three STP homologues in *A. flavus*. Sugar uptake assays in *A. flavus* and complementation in a hexose transport-deficient *S. cerevisiae* strain confirmed their transporter function. STP mutants were further tested in plant and animal models, revealing their roles in sugar acquisition, metabolism, and pathogenicity. These findings highlight the importance of STPs in *A. flavus* virulence and metabolism.

## Materials and methods

### Strains and culture conditions

*A. flavus* CA14Δ*ku70*Δ*pyrG* was used as the parental strain for transformation. CA14Δ*ku70* was used as the wild type (WT) for phenotypic analysis. WT, mutant and revertant (RT) stains were cultured on YG or Minimal medium (MM) (Armitt et al., 1976). Five millimoles of uracil and uridine were added for the strains with *pyrG* auxotrophy. The spores were collected by using 0.2% Tween-20 (v/v) from plates with 48 h of incubation at 37°C. Mycelia was harvested from liquid medium cultivation at 37°C with shaking at 200 rpm, washed with distilled water, frozen in liquid nitrogen, and grounded using mortar and pestle. The mycelium powder was stored at -80°C for RNA extraction. *S. cerevisiae* EBY.VW4000 strain was cultured in YPM or *S. cerevisiae* EBY.VW4000 harboring plasmid pRS424-EGFP was grown in the synthetic medium (SD) supplemented with drop-out amino acids lacking tryptophan (SD-Trp^-^), added 1% maltose and other carbon sources unless otherwise mentioned and incubated at 28°C.

### Construction of *A. flavus* sugar transporter mutant and revertant strains

The sugar transporter mutant Δ*1982*, Δ*5374* and Δ*9351* and RT strains were constructed by homologous recombination strategy. Each upstream and downstream flanking homologous arm (~1 kb) was generated by PCR. Likewise, the *pyrG* (~1.6 kb) fragment was PCR amplified from the pEXPYR plasmid. Three fused fragments were assembled in pCE-Zero vector. The deletion cassette was transferred to CA14Δ*ku70*Δ*pyrG* protoplasts. Transformants were screened on MM supplemented with 1 M sorbitol and then verified by PCR. For generation of RT constructs, RT constructing containing upstream flanking region, gene, pyrithiamine (PT) marker and *pyrG* were PCR amplified. PT fragment was PCR amplified from plasmid pPTRII. All the fragments were fuse-cloned to pCE zero vector and transferred to STP mutant protoplasts on MM screening plates supplemented with 0.1 µg/mL PT. The appeared colonies were screened and further PCR verified by primer pairs (**Supplementary Table S2**).

### Functional complementation of *A. flavus* STP genes in a hexose transport-deficient *S. cerevisiae* strain

The hexose transporter-deficient *S. cerevisiae* strain EBY.VW4000, which cannot grow on glucose but can grow on maltose, was used to validate the function of *A. flavus* STP genes. ORFs of *G4B84_001982* and *G4B84_005374* were amplified from *A. flavus* cDNA with *Bam*H I sites and cloned into the *Bam*HI-linearized yeast shuttle vector pRS424-EGFP (under the HXT7 promoter and terminator), generating plasmids pRS424-*1982*-EGFP and pRS424-*5374*-EGFP. Primer sequences are listed in **Supplementary Table S2**. These plasmids were transferred to EBY.VW4000 strain by electroporation, and transformants were selected on SD-Trp^-^ medium. Integration was confirmed by PCR of genomic DNA using gene and GFP-specific primers. Single colonies were tested for growth on SD-Trp^-^ medium with glucose. Cultures were grown to OD_600_ 0.8 - 1.0, washed with PBS, serially diluted (1:10), and spotted on SD-Trp^-^ plates containing different sugars. Plates were incubated for 3 days and photographed.

### Growth assay of *A. flavus* STP mutants on various carbon sources

MM supplemented with 1% of various sugars, glucose, N-acetyl-D-glucosamine (GlcNAc), glucosamine (GlcN), fructose, xylose, galactose, sucrose, maltose, mannose, arabinose, glycerol, or ethanol as the sole carbon source was prepared to assess sugar utilization by mutant strains. Fresh conidia from WT, mutant, and RT strains were serially diluted (10^5^-10²), and 10 μL aliquots were spot-inoculated onto the media. Plates were incubated at 37°C and photographed after two days.

### Colony growth and conidiation assays

Conidia from WT, mutant, and RT strains were point-inoculated at the center of MM plates and incubated at 37°C. Colony diameters were measured along the same axis every 24 h for 10 days to assess growth rates, with colony morphology photographed on day 3 and 10. For conidiation analysis, conidia were harvested, washed and resuspended by using 0.2% Tween-20 and centrifuged at 5,000 rpm. Two-fold serial dilutions were prepared, and conidia were counted using a hemocytometer under a microscope.

### Conidial germination assay

Conidia (1×10^5^ CFU/mL) from WT, mutant, and RT strains were inoculated into 200 µL of liquid MM in 96-well plates and incubated at 37°C. Spore germination was observed under a microscope at 8, 10, and 15 hours. For each time point, ten random fields of view were examined, and the number of germinated versus total spores was counted to calculate germination rates.

### Sclerotium formation assay

WT, mutant, and RT strains were point-inoculated at the center of YG plates and incubated at 28°C in the dark for 7 days. Sclerotia were counted, and conidia and mycelia were washed with 75% ethanol prior to imaging. Each assay was performed in triplicate, and statistical analysis was conducted to assess differences in sclerotium formation between strains.

### Stress sensitivity assays

Serially diluted conidia (10^5^-10²) from each strain were point-inoculated onto MM plates containing various stress agents and incubated at 37°C. Stress conditions included 1.2 M sorbitol, 0.8 M NaCl, and 0.6 M KCl (osmotic stress); 5 mM H_2_O_2_ (oxidative stress); and 50 µg/mL Congo Red (CR) and 100 µg/mL Calcofluor White (CFW) for cell wall stress. Plates were photographed after 2 days.

### Cell wall composition analysis

Cell wall analysis was performed with slight modifications to a previously described method (François, 2006). *A. flavus* mycelia were cultured in MM liquid medium at 37°C, 200 rpm for 48 h, harvested by filtration, and ground in liquid nitrogen. The resulting powder was treated with SDS-BME buffer (50 mM Tris, 50 mM EDTA, 2% SDS, 1 mM TCEP) and boiled for 40 minutes. Cell wall fractions were washed with Milli-Q water until foam disappeared and freeze-dried. Ten milligrams of dried wall material were hydrolyzed with 75 μL of 75% H_2_SO_4_ at room temperature for 3 hours, diluted to 2 N H_2_SO_4_ with 0.95 mL Milli-Q water, and boiled at 100°C for 4 hours. The hydrolysate was neutralized using Ba(OH)_2_, and BaSO_4_ precipitates were removed after overnight incubation at 4°C. The supernatant was analyzed for monosaccharide content by HPAEC-PAD using a CarboPac PA10 column with an AminoTrap guard column, eluted at 1 mL/min with 18 mM NaOH at room temperature.

### Virulence assay

Virulence assays in *Galleria mellonella* infection model were conducted according to previously described method (Ahamefule et al., 2020; Champion et al., 2016). To assess virulence in *G. mellonella*, larvae were divided into control (0.02% Tween-20), WT, mutant, and RT groups, with 90 larvae per group. Each larva was injected with 10 µL of 1×10^6^ CFU/mL conidial suspension into the hind proleg using a Hamilton syringe. Larvae were incubated at 37°C, and survival rate was recorded at 24-, 48-, and 72-h post-infection. Larvae were considered dead if immobile with signs of melanization or darkening.

### Peanut and corn seed infection assay

The pathogenicity of mutant strains on crop seeds was assessed as previously described (Yang et al., 2018). Uniform-sized fresh corn and peanut seeds were selected, and endosperm was removed using toothpicks to prevent germination and provide infection site. Seeds were surface-sterilized with 0.05% sodium hypochlorite for 3 min, followed by 75% ethanol for 1 min, and rinsed three times with sterile water. Sterile seeds were placed in 100 mL flasks and inoculated with 1×10^6^ CFU/mL conidia of each strain. After incubation at 28°C for 30 minutes, seeds were cultured in the dark for 6 days with constant humidity maintained by wet filter paper. Post-infection, seeds were transferred to 50 mL tubes containing 20 mL of 0.2% Tween-20 and shaken vigorously for 5 min to release conidia. Spore suspensions were serially diluted, and spores were counted using a hemocytometer. Each experiment was performed in triplicate and repeated three times.

### Aflatoxin extraction and detection

Aflatoxin was extracted from 500 µL of culture filtrate using an equal volume of chloroform. The organic layer was collected, evaporated at 70°C, and analyzed by thin-layer chromatography (TLC). A solvent system consisting of acetone: chloroform (1:9, v/v) was used. Aflatoxins were visualized under UV light at 365 nm.

### Statistical analysis

All statistical analyses were conducted using GraphPad Prism 8. Data are presented as mean ± standard deviation (SD). Two-group comparisons were analyzed using a Student’s t-test, while multiple comparisons were assessed using one-way ANOVA and pot hoc tests Dunnett’s was conducted.

## Results

### Identification, expression and structural analysis of putative STPs in *A. flavus*

A BLASTp search of the *A. flavus* genome using *S. cerevisiae* hexose transporters and sensors (Snf3 and Rgt2) identified approximately 100 candidate proteins, from which the top seven hits were selected for further analysis (**Supplementary Table S1**). These candidates, ranging from 513 to 672 amino acids in length, all belong to the STP subfamily of the MFS family.

**Figure 1:**
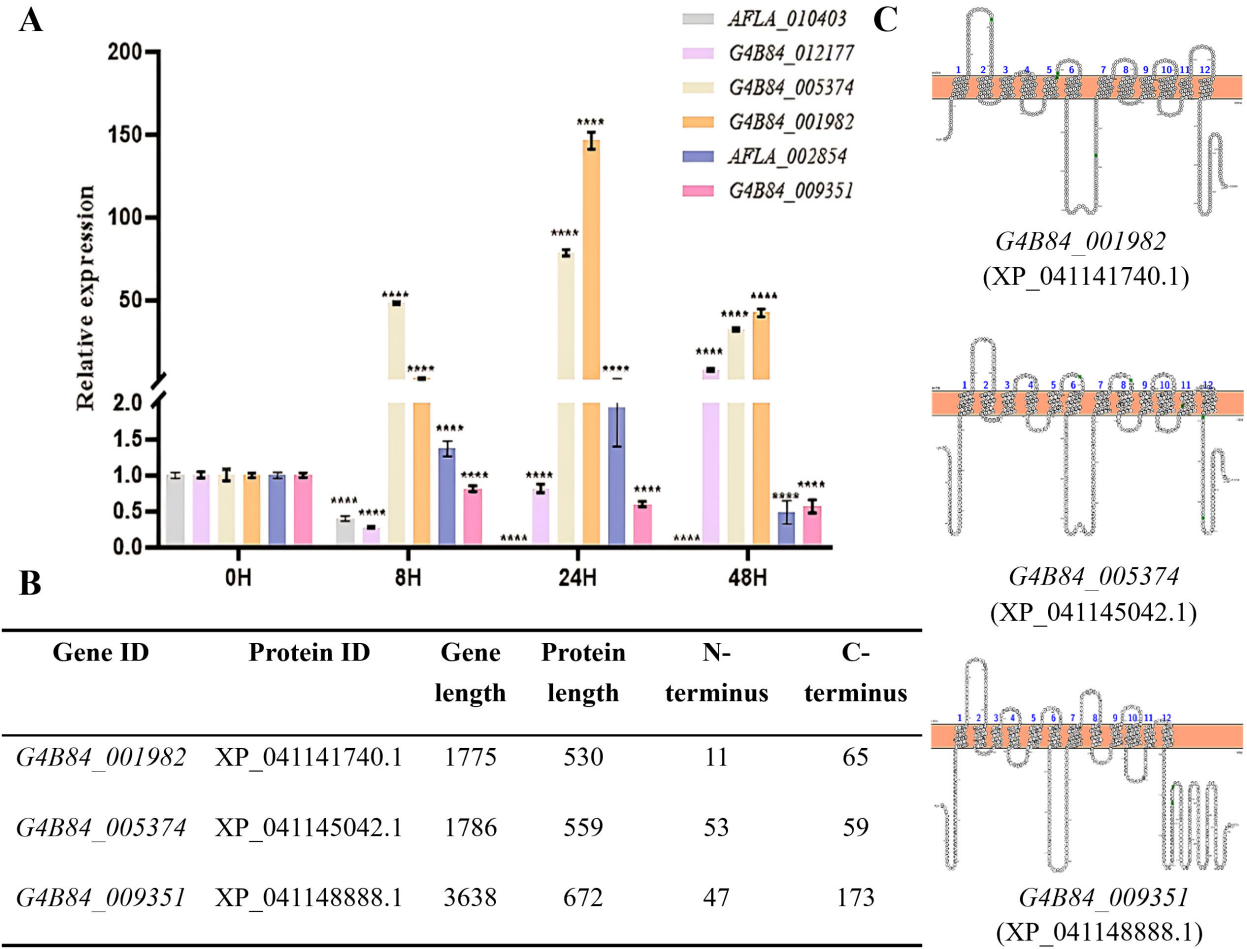
Transcriptional response, structural features, and topology of selected STPs in *A. flavus.* **(A)** RT-PCR analysis of putative STPs at different developmental stages: conidia (0 h), germination (8 h), hyphal growth (24 h), and exponential phase (48 h) in YG medium. *Tublin* served as the internal control, and expression levels were normalized to the 0 h stage. Relative expression was calculated using the 2^-ΔΔCt^ method, asterisks indicate significant differences from the control group (Student’s *t*-test, ****p < 0.0001). **(B)** Summary of selected STP candidates showing predicted N- and C-terminal lengths. **(C)** Predicted transmembrane topology of STPs based on TMHMM and PROTTER, indicating 12 α-helical transmembrane domains with cytoplasmic N- and C-termini.

To assess their expression dynamics, RT-PCR was performed at 0, 8, 24, and 48 h in the presence of 1% glucose (**Figure 1A**). Among the seven candidate genes, *G4B84_001982* showed the highest transcript levels across all stages, followed by *G4B84_005374*, which peaked during germination and hyphal growth. These patterns suggest that the corresponding MFS transporters may play important roles in fungal development and adaptation.

Phylogenetic analysis supported their functional classification, revealing that these transporters cluster with known fungal sugar transporters (**Supplementary Figure S1**). Specifically, XP_041141740.1 (encoded by *G4B84_001982*) grouped with *N. crassa* Rco-3 (a high-affinity glucose transporter), *T. reesei* Str1 (xylose transporter), *A. niger* XltA, and *A. nidulans* MstC, while XP_041145042.1 (encoded by *G4B84_005374*) and XP_041148888.1 (encoded by *G4B84_009351*) clustered with *C. graminicola* Hxt2, another high-affinity glucose transporter.

Structural analysis using TMHMM and PROTTER predicted XP_041141740.1, XP_041145042.1 and XP_041148888.1 harbored a conserved structure of 12 transmembrane α-helices with cytoplasmic N- and C-termini, a hallmark of MFS transporters (Saier, 1999). Intriguingly, while most candidates had short C-termini, XP_041148888.1 possessed an unusually long 173-amino-acid C-terminal tail, a typical feature of sugar sensors (**Figures 1B, C**), suggesting a potential sugar-sensing function in *A. flavus*.

Although MFS sugar transporters share low amino acid sequence similarity (12 - 18%), they possess a highly conserved architecture comprising 400 – 600 amino acid residues and 12 transmembrane (TM) helices arranged in N- and C-terminal domains that ensure efficient transport activity (Madej et al., 2014). For example, TMs 1, 4, 7, and 10 are critical for carbohydrate transport, with many residues directly interacting with substrates; TMs 2, 5, 8, and 11 link the N- and C-terminal domains and contribute to substrate binding and translocation; and TMs 3, 6, 9, and 12 provide structural stability. MFS transporters typically share the same three-dimensional fold and functional characteristics (Law et al., 2008; Taveira et al., 2024 (**Supplementary Figures S2A, B**).

Consistent with other hexose transporters, these proteins contain the Sugar Porter signature motifs, including the conserved D(N)RXGRR sequences between TM2-TM3 and TM8-TM9, and the PESPR motif at TM6 (Law et al., 2008). Most residues within these motifs are charged or polar, forming an extensive hydrogen-bond network that mediates interactions between the TM helices and the intracellular domains. Additionally, an aromatic residue–rich sequence (YFFYY) and the signature motif GR- - -G-G-G- - - - - -P-Y-SE-AP- -RG- - - - - -QL-TT-G (indicated by black bars in **Supplementary Figure S2C**) are conserved (Taveira et al., 2024). Notably, single point mutations of these conserved motif residues in bacterial homologs of glucose transporters (GLUT1 - 4) abolish transport activity entirely (Sun et al., 2012). Collectively, these structural and sequence analyses strongly suggest that *G4B84_001982*, *G4B84_005374* and *G4B84_009351* are functional sugar transporters or sensors involved in sugar uptake in *A. flavus*.

### STP in *A. flavus* are required for sugar metabolism, conidiation and germination

**Figure 2:**
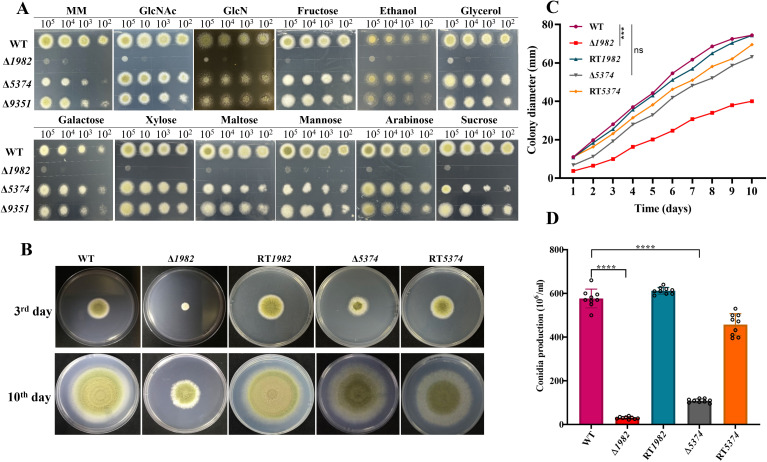
Growth phenotype of wild-type, mutant and revertant strains. **(A)** Sugar utilization assay of indicated strains on MM medium supplemented with 1% of the specified carbon sources. Serially diluted conidia (10^5^-10²) were spot-inoculated and incubated at 37°C for 2 days. **(B)** Colony morphology on MM after 3 and 10 days at 37°C. **(C)** Colony diameters were measured daily; data represent the mean ± SD of three biological replicates. Statistical significance was assessed by multiple t-tests (***P < 0.001; ns, not significant). **(D)** Conidial production after 10 days of incubation. Data are presented as mean ± SD; statistical significance was assessed by multiple t-tests (****P < 0.0001).

For function analysis, mutants of *G4B84_001982*, *G4B84_005374* and *G4B84_009351* were generated via gene replacement using *pyrG* as a selectable marker (**Supplementary Figure S3**). Sugar substrate specificity assays (**Figure 2A**) showed that the Δ*1982* mutant exhibited severe growth defects in the presence of all tested hexoses (glucose, fructose, sucrose, mannose, and maltose), pentoses (xylose and arabinose), and amino sugars (GlcNAc and GlcN). Additionally, Δ*1982* failed to grow on non-fermentable carbon sources such as ethanol and glycerol, indicating that this transporter may be involved in the uptake of a broad range of sugars and carbon sources.

The Δ*5374* mutant displayed markedly reduced colony growth and an albino phenotype on monosaccharides such as glucose, fructose, and mannose, as well as the disaccharide sucrose. However, its growth was not affected by alternative carbon sources, suggesting a selective, yet biologically significant role in sugar transport.

In contrast, the Δ*9351* mutant exhibited only minor defects in colony expansion and conidiation, with no substantial growth impairment across all the tested sugars. Despite its long C-terminal region - a signature of sugar sensors – the deletion of *G4B84_009351* did not result in a notable phenotype under these conditions. Consequently, subsequent analyses focused on the two mutants with pronounced phenotypic alternations: Δ*1982* and Δ*5374*.

To evaluate the impact of these mutations on fungal growth, colony diameters were compared among the strains (**Figures 2B, C**). The Δ*1982* displayed the most pronounced growth defect at 72 h and persisted throughout the observation period. Although Δ*5374* grew better than Δ*1982*, both mutants displayed rough, irregular colonies lacking the radial symmetry and smooth margins observed in the WT strain. Furthermore, the condition was significantly impaired in both mutants (**Figure 2D**). Collectively, these findings suggest that the STP genes, particularly *G4B84_001982* and *G4B84_005374*, play essential roles in sugar utilization, growth, and asexual development in *A. flavus*.

**Figure 3:**
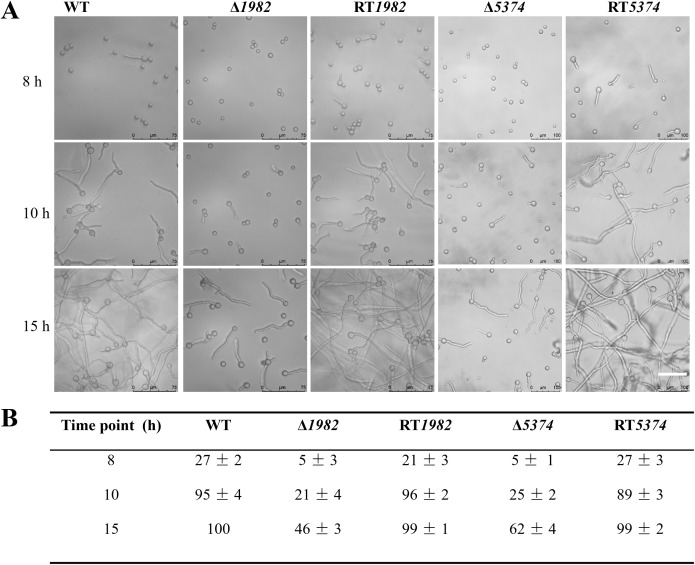
Germination dynamics of wild-type, mutant, and revertant strains in liquid MM. **(A)** Germination was monitored using differential interference contrast (DIC) microscopy (Leica) after static incubation at 37°C for 8 h, 10 h, and 15 h. Representative images are shown. Scale bar: 75 µm. **(B)** Germination rates were quantified by counting approximately 100 conidia per strain at each time point. The experiment was performed in triplicate, and data are presented as mean ± SD.

In liquid MM medium, STP mutant strains exhibited delayed germination compared to the WT strain. At 8 h, WT conidia had begun to swell and initiate germ tube formation, whereas the mutant strains had not yet reached the swelling stage. By 10 h, small hyphal extensions were observed in the WT, with a complete hyphal network established by 15 h, marking the transition to active mycelial growth. In contrast, the mutant strains showed significantly reduced germination rates, with no observable hyphal network formation. Specifically, Δ*1982* exhibited less than 50% germination, while Δ*5374* reached only 62%, compared to 100% germination in both WT and RT strains. These results demonstrate that deletion of STP genes significantly impairs conidial germination and early hyphal development in *A. flavus* (**Figures 3A, B**).

### Heterologous expression and functional characterization of *A. flavus* STPs in *S. cerevisiae* EBY.VW4000 strain

**Figure 4:**
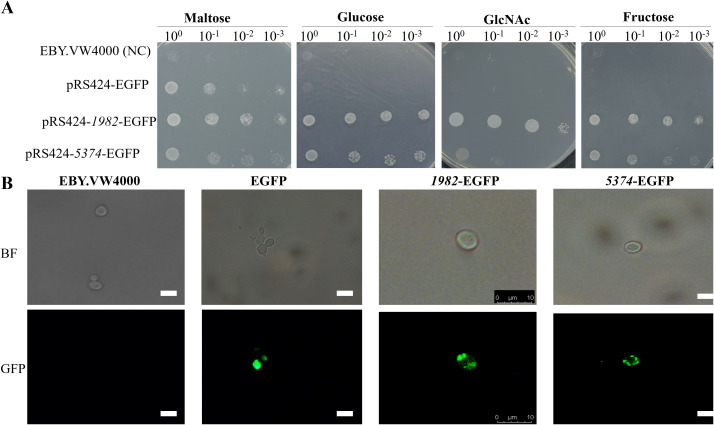
Subcellular localization and functional transport analysis of *A. flavus* STPs *1982* and *5374* expressed in yeast cells. **(A)** Functional complementation assay of EBY.VW4000 expressing *1982* or *5374* or harboring the empty vector (negative control). Serial 10-fold dilutions of log-phase cells were spotted onto SD-Trp^-^ agar plates supplemented with indicated sugars and incubated at 28 °C for 72 h to assess sugar transport capability. **(B)** Subcellular localization of STPs in *S. cerevisiae* strain EBY.VW4000. The coding regions of *1982* and *5374* were fused to the pRS424-EGFP vector and transformed into EBY.VW4000. Transformants were grown in SD-glucose medium, and localization was observed using fluorescence microscopy. Scale bar: 10 μm.

To investigate the functional roles of *A. flavus* STPs, the coding sequences of *G4B84_001982* and *G4B84_005374* were cloned into the yeast expression vector pRS424-EGFP and subsequently transformed into the *S. cerevisiae* hexose transporter-deficient strain EBY.VW4000. Confocal microscopy confirmed that both STPs were correctly localized to the plasma membrane in yeast (**Figure 4B**). Transformants were selected on maltose medium, which served as a positive control, while the strain carrying the empty vector served as a negative control.

Drop assay on SD-Trp^-^ plates revealed that expression of *1982* and *5374* restored the growth of EBY.VW4000 on glucose, confirming their function as glucose transporters (**Figure 4A**). Notably, strain *1982* also supported growth on fructose, GlcNAc, sucrose, mannose, xylose, and galactose, indicating broad substrate specificity. In contrast, strain *5374* facilitated growth on sucrose and mannose, suggesting a more limited but overlapping sugar transport capability (**Figure 4** and **Supplementary Figure S5**). These findings demonstrate that the two STPs mediate uptake of multiple sugar substrates when expressed in yeast.

### Deletion of STPs compromised cell wall integrity and stress response

**Figure 5:**
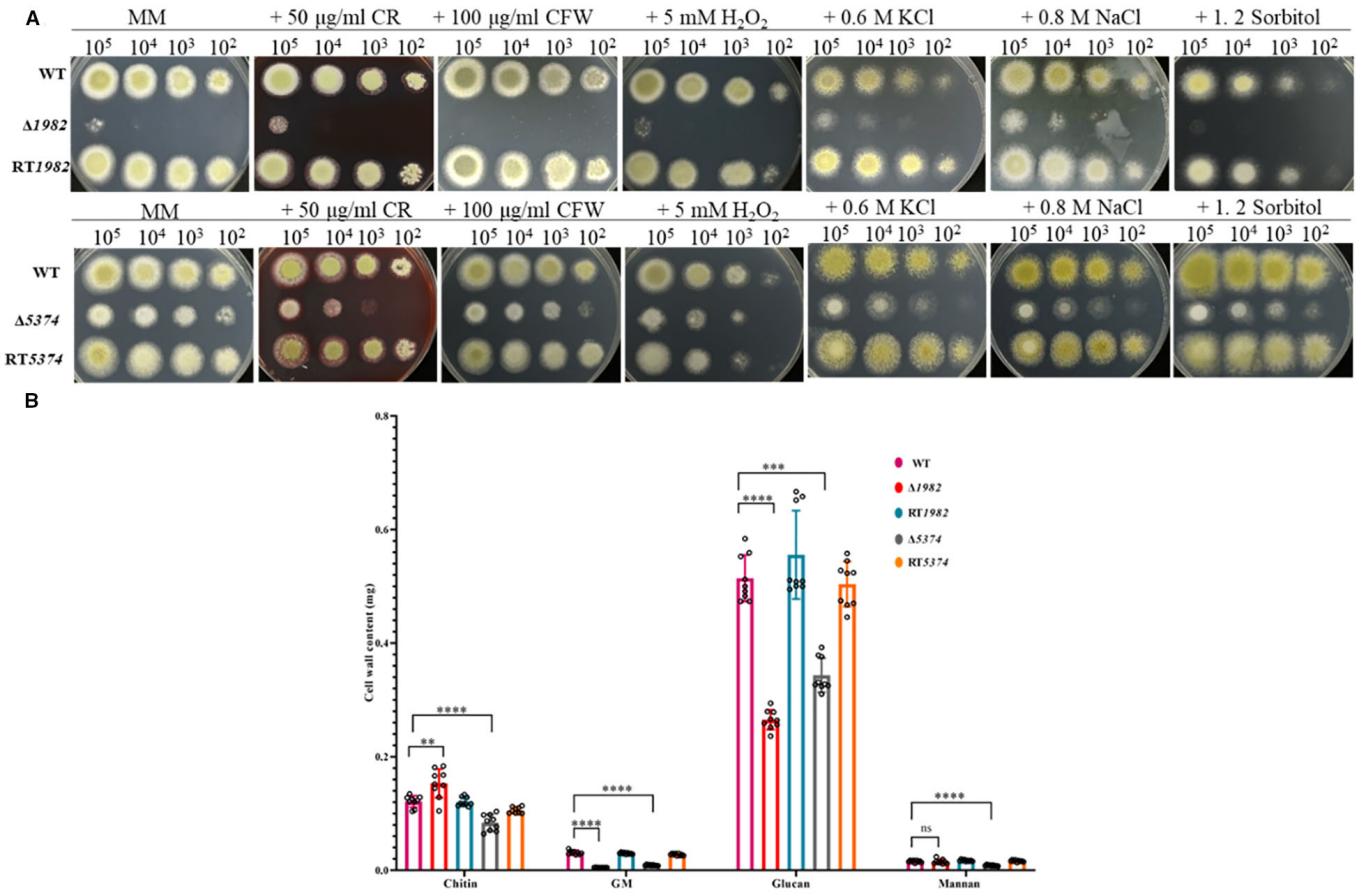
Sensitivity of wild-type, mutant, and revertant strains to cell wall, osmotic, and oxidative stresses. **(A)** Growth phenotypes on MM plates supplemented with cell wall stressors, osmotic and oxidative stress agents after 48 h at 37°C. **(B)** Quantification of cell wall components from 10 mg dry mycelia after 48 h cultivation in liquid MM (2 × 10^8^ conidia/200 mL). Data represent mean ± SD from three biological replicates; significance was assessed using t-tests (****P < 0.0001; ***P < 0.001; **P < 0.01; ns, no significance).

Since sugars are key precursors for cell wall polysaccharide biosynthesis, we investigated whether disruption of STP would affect the cell wall integrity (CWI) of *A. flavus*. As shown in **Figure 5A**, the growth of the mutants was markedly inhibited in the presence of CR and CFW. To quantitatively assess CWI defects, we measured the cell wall components of each strain in glucose-grown cultures (**Figure 5B**). Compared to the WT strain, both Δ*1982* and Δ*5374* showed significant reductions in glucan (by 50% and 35%, respectively) and galactomannan (by 83% and 70%, respectively) content. Interestingly, Δ*1982* exhibited a 42% increase in chitin content, while Δ*5374* showed a 27% decrease. Mannan content was slightly reduced in Δ*1982* (4%) but decreased substantially in Δ*5374* (46%). The observed reduction in glucan content aligns with the CR sensitivity of both STP mutants (**Figure 5A**), further supporting our finding that two STPs are engaged in maintaining CWI in *A. flavus*.

Given the observed CWI defects, we further assessed the sensitivity of STP mutants under various environmental stress conditions. As shown in **Figure 5A**, both Δ*1982* and Δ*5374* mutants exhibited pronounced sensitivity and significantly impaired growth under ionic (0.8 M NaCl, 0.6 M KCl) and non-ionic (1.2 M sorbitol) osmotic stress, as well as oxidative stress induced by H_2_O_2_. In contrast, the WT strain maintained normal growth under these conditions. The higher inhibition rates observed in the mutant strains indicate that these STP genes are critical for mediating resistance to osmotic and oxidative stress. These findings suggest that STP-mediated sugar uptake plays an essential role in enabling *A. flavus* to adapt to hostile environments, potentially contributing to its survival and pathogenicity under adverse conditions.

### Deletion of STP genes affects sclerotium production

**Figure 6:**
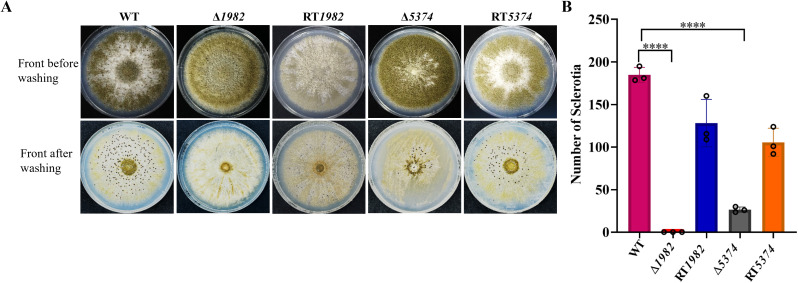
Sclerotium formation in wild-type, mutant, and revertant strains. **(A)** 10^5^ conidia of each strain were inoculated on YG plates and incubated in the dark at 28°C for 7 days to induce sclerotia formation. **(B)** Sclerotia were quantified, and values are presented as mean ± SD. Statistical significance was determined using multiple t-tests (****P < 0.0001).

The role of STPs in sclerotia formation was further examined. The Δ*1982* showed a near-complete abolition of sclerotia formation, and Δ*5374* produced very few sclerotia, both before and after ethanol wash, while abundant sclerotia were observed in the WT and RT strains (**Figure 6A**). Quantitatively, Δ*1982* and Δ*5374* exhibited 99% and 85% reductions in sclerotia production, respectively (**Figure 6B**). These findings suggest that STP deletion impairs *A. flavus* adaptation to adverse conditions, consistent with their heightened sensitivity to chemical and osmotic stresses.

### Deletion of STPs impaired crop seed colonization and aflatoxin production

The above findings highlight the critical role of STP genes in growth, conidiation, sclerotia formation, and stress responses-key factors for survival, host invasion, and pathogenicity. We therefore hypothesized that deletion of STPs may compromise the pathogenicity of *A. flavus*.

**Figure 7:**
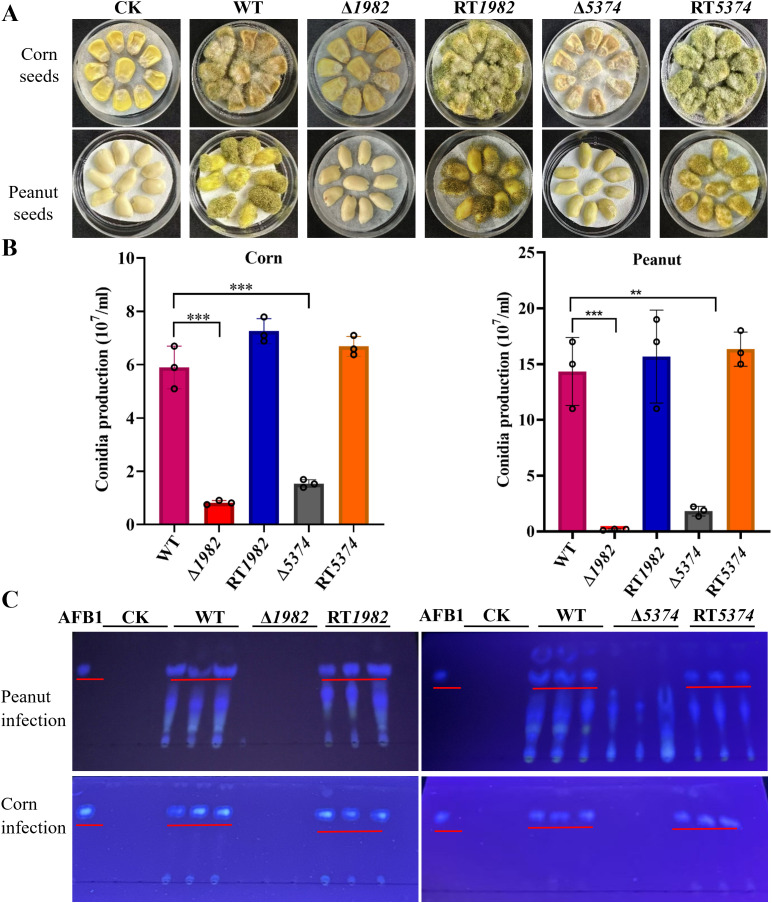
Colonization of wild-type, mutant and revertant strains on crop seeds. **(A)** 10^6^ conidia of indicated strains were inoculated on peanut or corn seeds and incubated at 28 °C for 6 days in the dark. Tween-20 was used as control (CK). **(B)** Conidia washed from the infected peanut or corn seeds were counted using a hemocytometer. Values represent means ± SD from three biological replicates with triplicate setup. Asterisks indicate significant differences (**P < 0.01, ***P < 0.001). **(C)** TLC analysis of AFB1 extracted from spore suspensions washed from peanut and corn seed surfaces using equal volumes of chloroform. Chloroform alone was used as control (CK).

After 6 days of inoculating corn and peanut seeds with WT, mutants, and RT strains, seed colonization by Δ*1982* and Δ*5374* was visibly reduced compared to WT and RT strains (**Figure 7A**). Conidia recovered from infected seeds confirmed a significant reduction in spore production by the mutants (**Figure 7B**). We hypothesized that this colonization defect might result from the mutants inability to utilize seed-derived nutrients. To test this, growth assays were performed on corn or peanut powder agar plates. Δ*1982* failed to grow on peanut powder, and Δ*5374* showed limited growth on corn powder, supporting the notion that these mutants are nutritionally impaired (**Supplementary Figure S4**).

*A. flavus* produces AFB1 which poses significant threat to humans, animals and plants. Furthermore, keeping the important physiological and cellular role of STP genes in *A. flavus*, we analyzed the amount of AFB1 accumulated in the infected seeds. TLC analysis revealed that AFB1 was only accumulated in the seeds infected by the WT and RT strains, whereas no AFB1 was detected in the seeds infected by Δ*1982* and Δ*5374* (**Figure 7C**). These results clearly demonstrate that the deletion of STPs resulted in its inability to colonize and grow on crop seeds, and AFB1 could not be accumulated in the mutant infected crops. Therefore, studying and targeting *A. flavus* STP genes more in depth might be a practical strategy to reduce aflatoxin contamination.

### Deletion of STPs leads to attenuated virulence in the *Galleria mellonella* infection model

**Figure 8:**
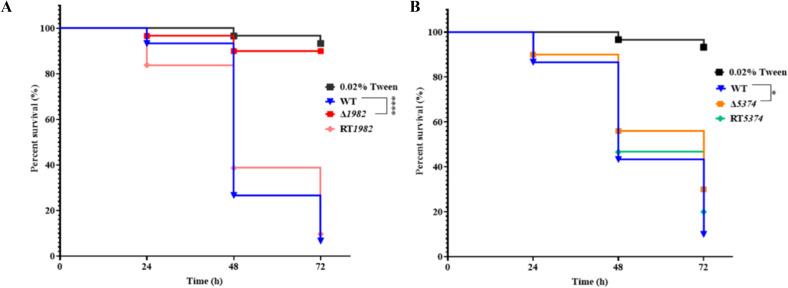
Virulence assay of wild-type, mutant and revertant strains in *G. mellonella*. **(A)** Kaplan-Meier survival curves of larvae infected with Δ*1982* conidia; **(B)** Kaplan-Meier survival curves of larvae infected with Δ*5374* conidia. Survival was monitored at 24-, 48-, and 72-h post-infection. Larvae treated with 0.02% Tween-20 served as the control. Experiments were performed at 37°C in three biological replicates with triplicate setup.

*A. flavus* is a major cause of invasive aspergillosis and superficial infections, relying on sugar acquisition from the host to establish infection. To assess the role of STPs in virulence, *G. mellonella* larvae were infected with WT, STP mutants, and RT strains, and survival rates were monitored over 72 h using Kaplan-Meier analysis (**Figure 8**). The mutants showed significantly reduced virulence compared to WT and RT strains. At 48 h post-infection, over 50% mortality was observed in larvae infected with WT or RT strains, whereas mortality rate was only 44% for Δ*5374* and only 10% for Δ*1982*. By 72 h, survival rates were 6 - 10% for WT, but remained high at 90% and 30% for Δ*1982* and Δ*5374*, respectively. These results indicate that STP genes are important for *A. flavus* pathogenicity in this model.

## Discussion

*A. flavus* represents a dual threat to both human health and agricultural security. This opportunistic pathogen causes invasive aspergillosis, commonly manifesting as pulmonary infections and invasive rhinosinusitis (Rudramurthy et al., 2019). Equally concerning is its production of AFB1, a notorious hepatocarcinogen inducer that contaminates staple crops, posing serious threats to food safety, public health, and global trade (Felizardo and Câmara, 2013). Effective strategies to mitigate aflatoxin contamination are urgently needed.

Sugars, particularly glucose, are vital energy sources that drive fungal growth, metabolism, and virulence. Glucose metabolism through glycolysis and the pentose phosphate pathway is closely linked to cell wall biosynthesis and fungal pathogenicity (Fleck et al., 2011). Recent studies also highlight the role of glucose homeostasis in host defense against fungal infections (Tucey et al., 2020) (Weerasinghe and Traven, 2020). Therefore, maintaining glucose homeostasis is crucial for preventing severe fungal diseases (Tucey et al., 2018). Fungi rely on membrane STPs to import sugars, regulating intracellular metabolism (Perlin et al., 2014). While most knowledge on fungal STPs derives from *S. cerevisiae*, where hexose uptake occurs via diffusion, STPs in pathogenic fungi like *U. maydis*, *Colletotrichum* spp., *C. albicans*, and *C. neoformans* have been linked to growth and virulence (Wittek et al., 2017) (Schuler et al., 2015) (Pereira et al., 2013) (Chen et al., 2019) (Brown et al., 2006) (Alvarez and Konopka, 2007) (Liu et al., 2013). These plasma membrane MFS transporter proteins play critical roles in signaling, metabolism, development, and infection, making them promising antifungal targets.

In this study, we identified three candidate STPs in the *A. flavus* genome (*G4B84_001982*, *G4B84_005374*, and *G4B84_009351*) based on conserved MFS domains and expression profiling (**Figure 1**, **Supplementary Figures S1**, **S2**, **Supplementary Table S1**). Functional analyses of mutants revealed that deletion of G4B84_001982 severely impaired growth on multiple sugars, suggesting it functions as a primary hexose transporter in *A. flavus* (**Figure 2A**). The broad substrate specificity observed here differs from that of previously reported STPs, as it was unexpected that deletion of a single STP, despite the presence of other transporters in *A. flavus*, resulted in severe growth defects. In *S. cerevisiae*, comparable defects typically require simultaneous deletion of multiple hexose transporters, or the deletion of either SNF3 or RGT2, which serve as sensors regulating the expression of several transporters (Boles and Hollenberg, 1997; Reifenberger et al., 1997). These findings suggest that G4B84_001982 may function as a major hexose transporter in *A. flavus*. Δ*5374* showed substrate-specific defects on glucose, maltose, fructose, mannose, and sucrose, indicating metabolic flexibility via diverse sugar uptake pathways. No single STP was found to be exclusively responsible for only one sugar, likely due to functional redundancy.

STPs are essential for fungal growth, development, and virulence, as demonstrated in other fungi such as *Verticillium dahliae*, *Penicillium digitatum*, and *Neurospora crassa* (Chen et al., 2023; Madi et al., 1997; Nakaune et al., 2002). Similarly, Δ*1982* and Δ*5374* in *A. flavus* exhibited reduced growth, delayed germination, and altered colony morphology. These findings confirm that STPs are essential for nutrient acquisition and metabolism, and that their deletion disrupts cellular metabolism and energy production, thereby impairing key physiological processes in *A. flavus* (**Figures 3B-D**, **4**). The fungal cell wall, composed mainly of glucans, chitin, and galactomannan, is a dynamic structure crucial for pathogenicity and stress adaptation (Adams, 2004). Like *M. oryzae* STP mutants that resulted in reduced soluble saccharides and sugar utilization defects (Chen et al., 2023), *A. flavus* Δ*1982* and Δ*5374* mutants also displayed hypersensitivity to cell wall stress agents (CR and CFW) and altered cell wall composition (**Figure 5**). Notably, Δ*1982* showed reduced glucan and galactomannan but increased chitin, a compensatory response known in *S. cerevisiae* to maintain cell wall integrity (Nakaune et al., 2002).

MFS proteins function are known to mediate stress tolerance and contribute to drug resistance (Liu et al., 2021; Nakaune et al., 2002). Δ*Cg*MFS1 mutant displayed significantly increased sensitivity to H_2_O_2_, indicating a key role for *Cg*MFS1 in the oxidative stress response (Liu et al., 2021). Correspondingly, *A. flavus* STP mutants exhibited heightened sensitivity to oxidative and osmotic stresses and were unable to form normal sclerotia-structures that serve as reservoirs for sexual spore production and are critical for long-term survival (**Figures 6C** and **7**). Deletion of STPs likely restricted sugar availability and may have impaired trehalose-6-phosphate metabolism (Wang et al., 2018), which is important for sclerotia development, a hypothesis that warrants further metabolic profiling.

During host infection, nutrient competition is intense. Pathogens secrete cell wall degrading enzymes (CWDEs) to access plant sugars for growth and invasion. In seed infection assays, Δ*1982* and Δ*5374* mutants failed to colonize corn and peanut seeds effectively, correlating with reduced conidial production and loss of AFB1 synthesis (**Figure 7**). This is likely due to impaired sugar uptake disrupting metabolic pathways and downregulating CWDE expression, which limits hyphal development and host penetration. Additionally, compromised cell wall integrity in mutants enhances host resistance. Virulence assays in *G. mellonella* further confirmed attenuated pathogenicity of STP mutants, underscoring their critical role in both plant and animal infections (**Figure 8**). While complementation of Δ*5374* restored most phenotypes, incomplete recovery of conidiation and sclerotia formation suggests additional regulatory layers or epigenetic factors influencing these developmental processes.

The ongoing evolution of plant pathogens underscores the urgent need for resistant cultivars, with molecular breeding tools increasingly targeting key infection mechanisms such as sugar acquisition (Savadi et al., 2018). Sugar transport proteins (STPs), conserved across pathogenic fungi, represent promising broad-spectrum antifungal targets (Lata et al., 2023). However, selective inhibition is complicated by their similarity to host transporters. At the plant-pathogen interface, competition for extracellular sugars is a critical infection determinant, where fungal high-affinity transporters-like the corn smut *Um*SRT1-can outcompete host counterparts (Wahl et al., 2010). High-resolution structural studies of STPs (Bavnhøj et al., 2021, 2023) and computational modeling tools (Pajak et al., 2019; Schiffman and Rother, 2013) facilitate identification of key residues and motifs essential for function, as shown by mutation studies in GLUT homologs that disrupt transport activity (Sun et al., 2012).

Engineering STPs offers a viable path for durable resistance, exemplified by the wheat Lr67res hexose transporter variant conferring broad fungal resistance through altered carbon partitioning and defense priming (Milne et al., 2019). Additionally, targeted use of sugar analogs or natural inhibitors at infection sites could block fungal sugar uptake without harming hosts (Lemoine and Delrot, 1987; Pajak et al., 2019; Palmer et al., 2015; Schiffman and Rother, 2013). Host-induced gene silencing (HIGS) of fungal STPs, as demonstrated in *Verticillium dahliae*, further supports this strategy (Wang et al., 2016; Zhang et al., 2016). Future research must elucidate regulatory networks controlling STP expression during infection, requiring coordinated efforts from multidisciplinary teams to advance STP-targeted approaches for managing fungal diseases and aflatoxin contamination in agriculture.

## Data Availability

The datasets presented in this study can be found in online repositories. The names of the repository/repositories and accession number(s) can be found in the article/**Supplementary Material**.
