# Supplementary material for: Key sugar transporters drive development and pathogenicity in Aspergillus flavus
Source: Front Cell Infect Microbiol. 2025 Sep 8;15:1661799. doi: 10.3389/fcimb.2025.1661799 (PMC12450973; doi:10.3389/fcimb.2025.1661799)
Supplement: Supplementary file 1 [file DataSheet1.docx]

Key sugar transporters drive development and pathogenicity in *Aspergillus flavus*

**Raheela Yasin^1,2^, Sayed Usman^1,3^, Qijian Qin^1^, Xiufang Gong^1^, Bin Wang^1^, Cheng Jin^1,3*^ and Wenxia Fang^1,3*^**

^1^Institute of Biological Sciences and Technology, Guangxi Academy of Sciences, Nanning, Guangxi, China.

^2^College of Life Science and Technology, Guangxi University, Nanning, Guangxi, China.

^3^State Key Laboratory of Mycology, Institute of Microbiology, Chinese Academy of Sciences, Beijing, China.

*Corresponding author’s email: wfang@gxas.cn or jinc@im.ac.cn


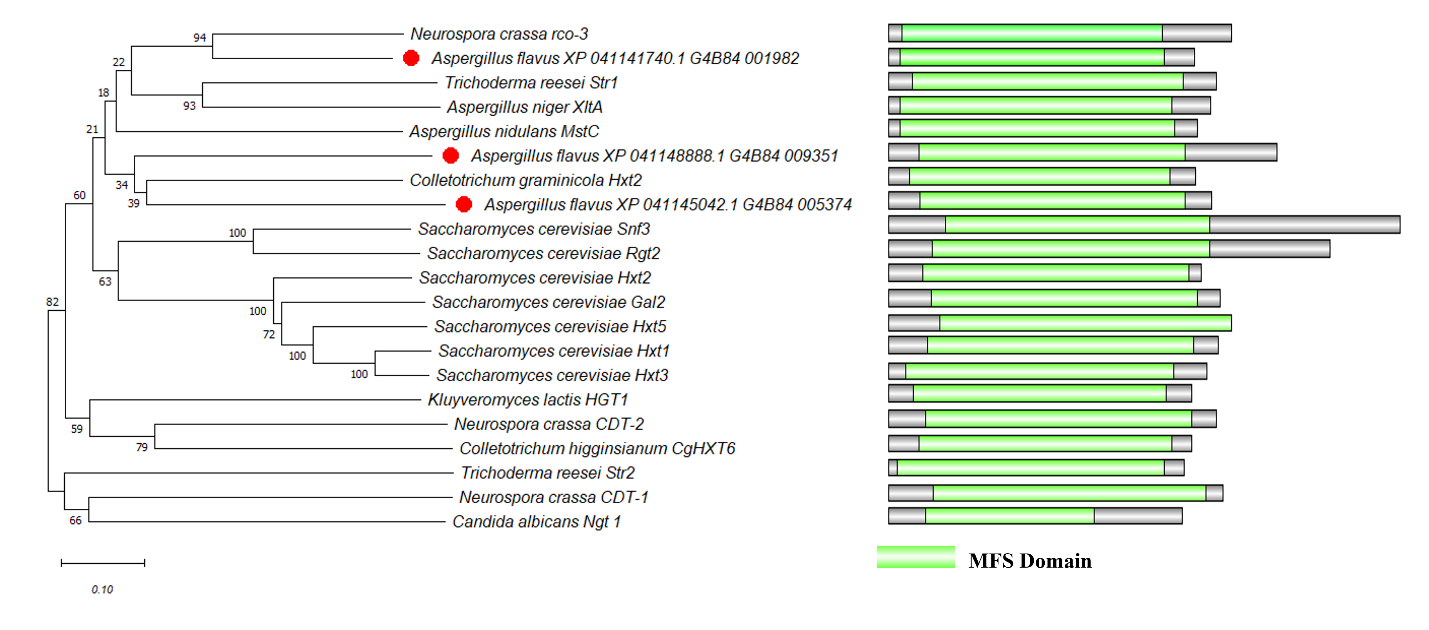


**Figure S1**. Phylogenetic relationship and protein domain analysis of STP orthologs in selected species. The MEGA software was used to conduct phylogenetic tree analysis of selected genes. The phylogenetic tree was constructed using the neighbour-joining method based on the full length of the ortholog proteins, with the number of bootstrap replicates set to 1,000. Protein domain analysis was performed using the conserved domain search function on the NCBI website, and the protein domains were visualized using IBS software.


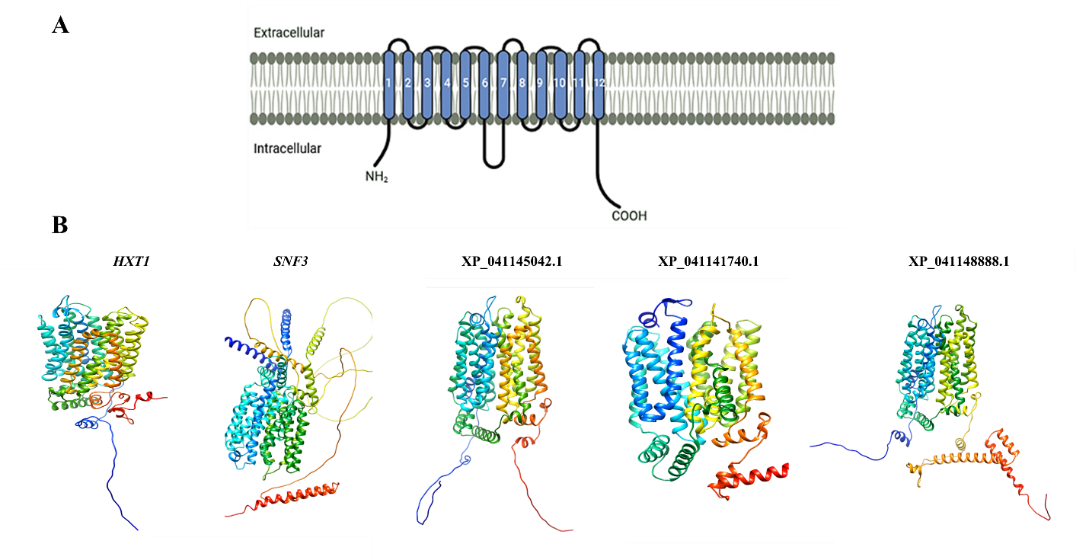

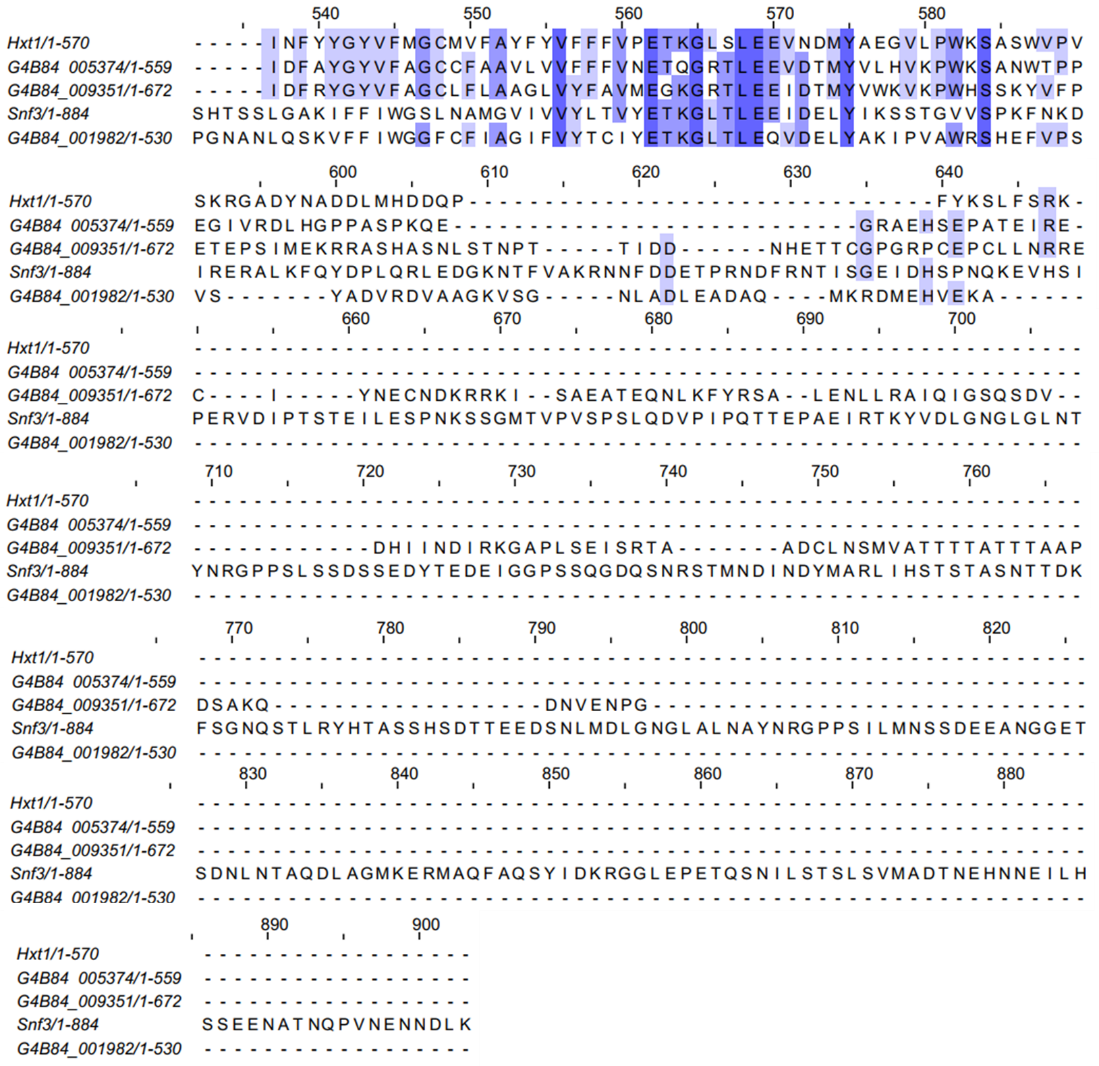

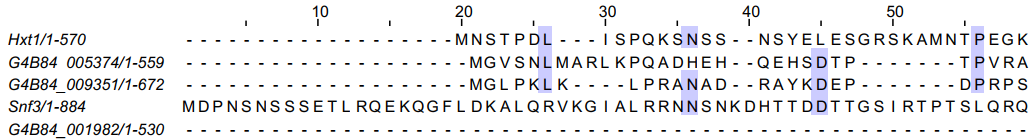

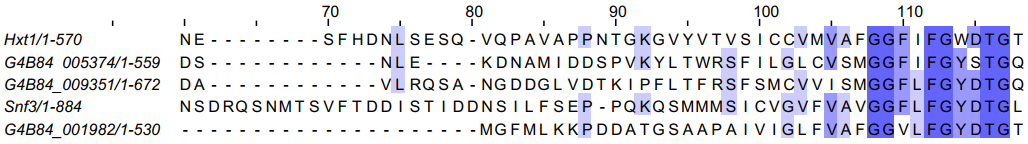

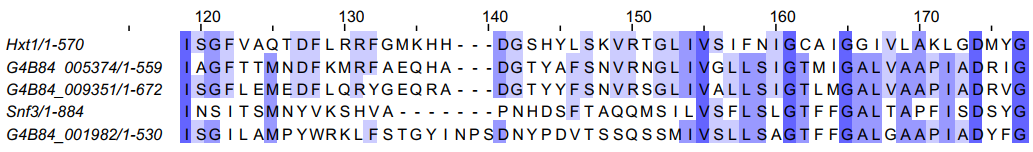

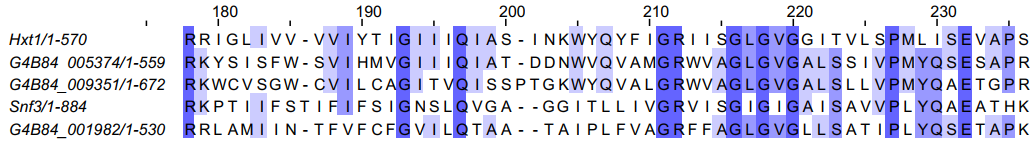

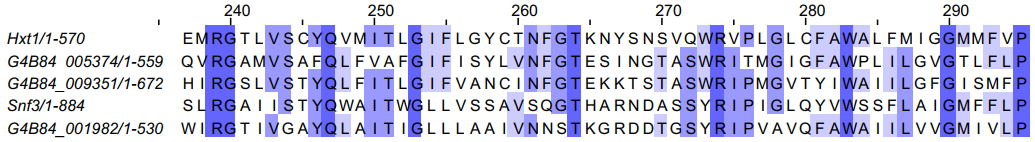

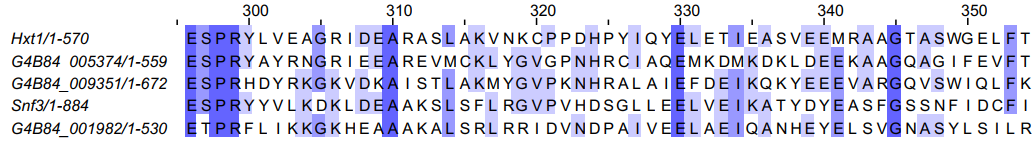

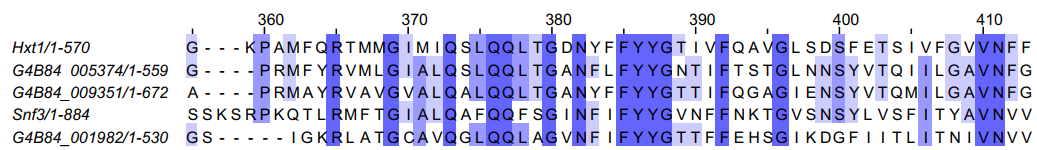

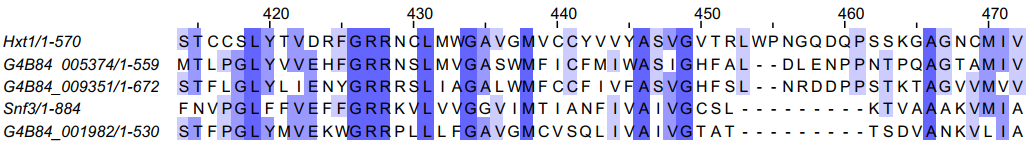

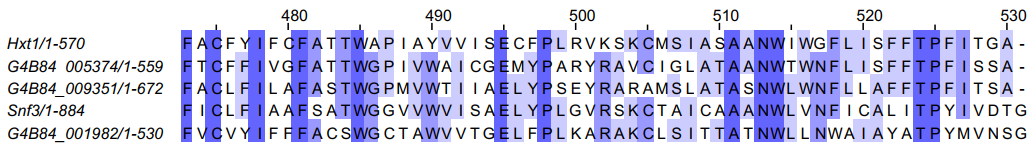


**C**

**Figure S2.** The MFS transporters are structurally and mechanistically conserved.

(A) Schematic representation of the canonical MFS topology showing the transmembrane (TM) helices of the N- and C-terminal domains. (B) Structural comparison of yeast Hxt1, Snf3, and *A. flavus* XP_041141740.1, XP_041145042.1, and XP_041148888.1. The N-terminus is shown in blue and the C-terminus in red. Models were generated using AlphaFold3. (C) Multiple sequence alignment of Hxt1, Snf3, and *A. flavus* STPs, performed with ClustalW and visualized in Jalview 2.11.2.6.


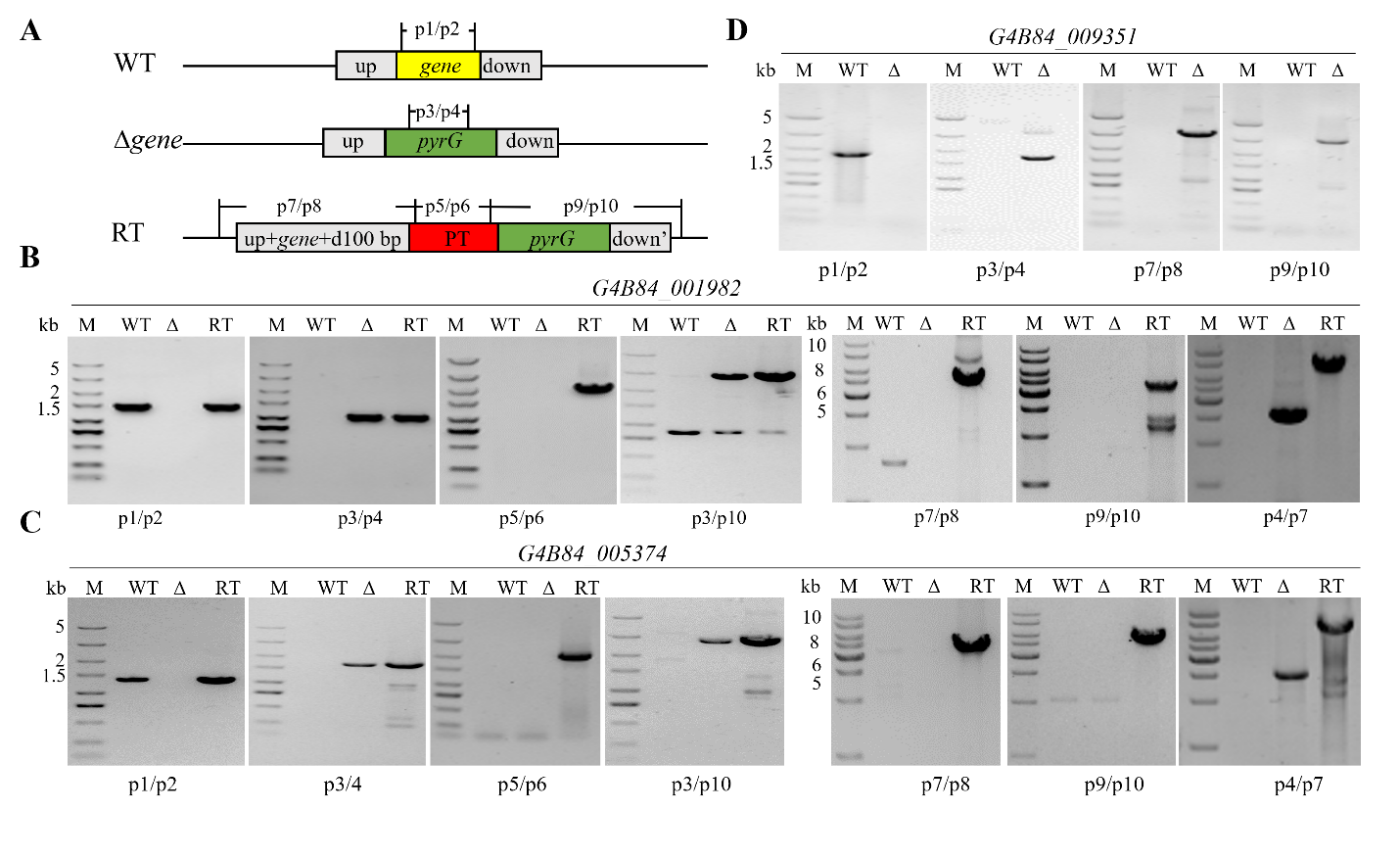


**Figure S3**. Construction and confirmation of the STP mutant and RT strains. **(**A) Homologous recombination strategies to generate the STP mutant and RT strains. PCR analysis of the Δ*1982* and RT*1982* strains (B), Δ*5374* and RT*5374* strains (C), Δ*9351* strains (D) using five pairs of primers as shown in Table S1.


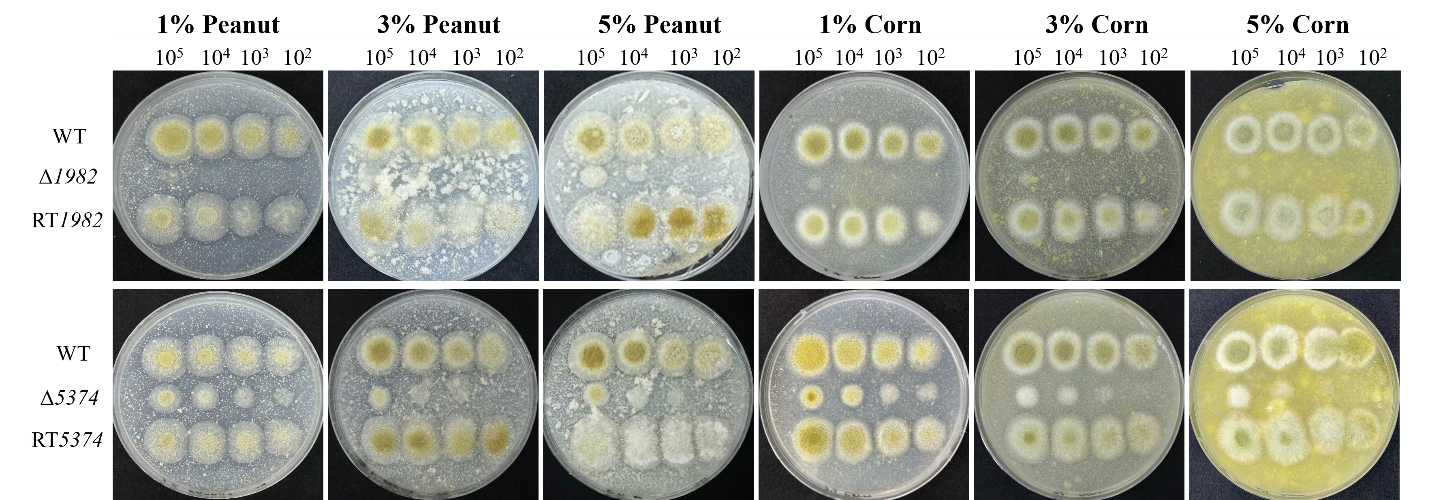


**Figure S4**. Growth analysis of wild-type, mutant and RT strains on corn and peanut powder at 1%, 3% and 5%. Serially diluted conidia 10^5^ - 10^2^ of each strain were inoculated on corn and peanut plates, incubated at 30^°^C for 2 days.

**
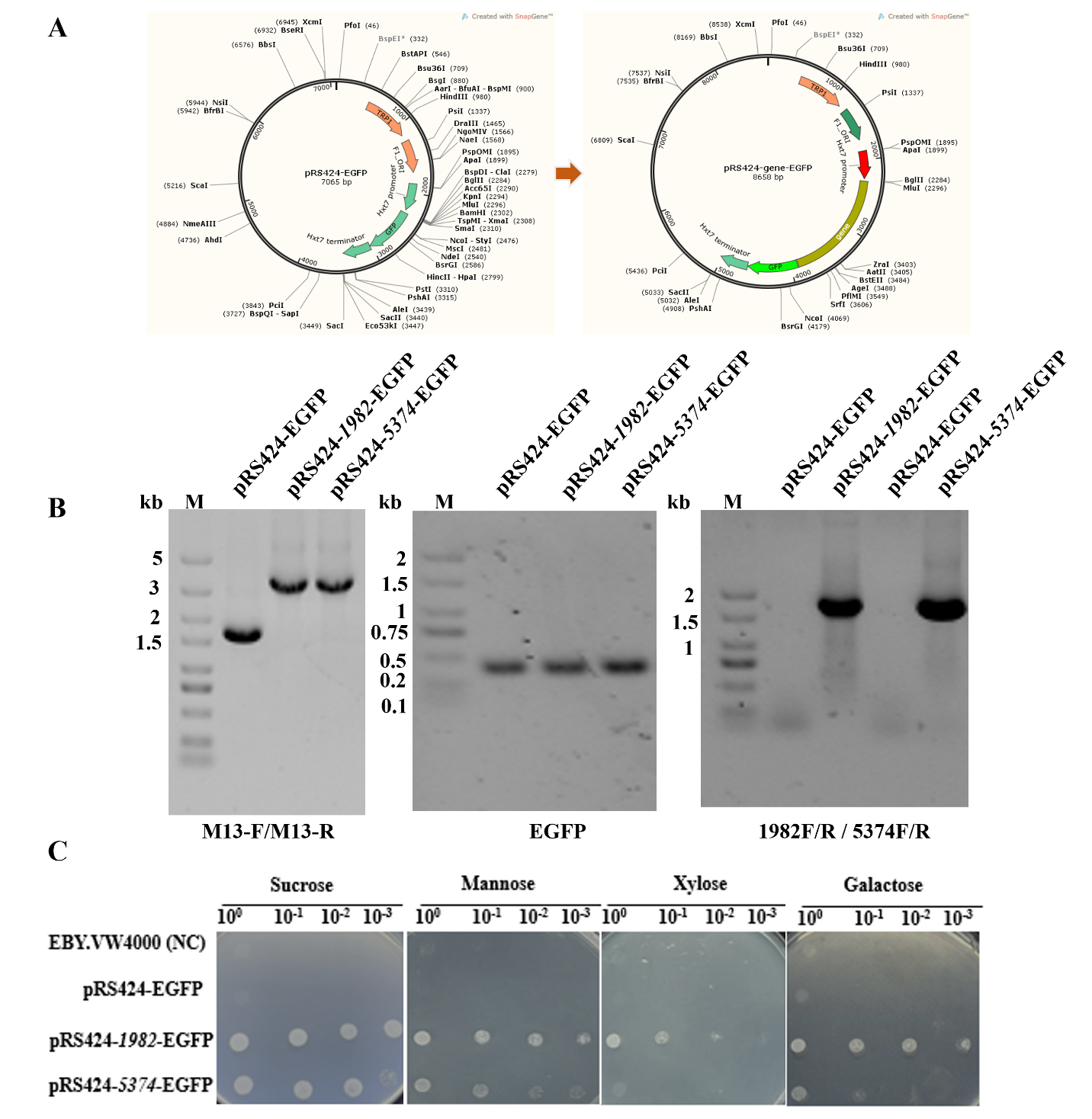
**

**Figure S5**. Construction, verification and growth assay of the yeast expression vector containing *A. flavus* STP CDS for complementation assay. (A) PCR validation of expression vectors by M13-F/R, EGFP primer pair and *1982*, *5374* insertion primer pair, respectively. pRS424-EGFP served as the negative control. (B) Growth of strain EBY.VW4000 containing the *G4B84_001982* or *G4B84_005374* or harboring the empty expression vector as negative control is indicated on sucrose, mannose, xylose and galactose. Serial dilutions of logarithmically growing cells were spotted onto SD-Trp^-^ agar plates and incubated at 28^°^C for 72 h.

**Table S1. *A. flavus* putative hexose transporters identified as possible homomogues of *S. cerevisiae*.**

| Serial number | Accession no. | Identity  (%) | Amino acid  (aa) | C-Terminus (bp) | Conserved Domains |
| --- | --- | --- | --- | --- | --- |
| 1 | *AFLA_010403* | 47.11 | 561 | 62 | MFS |
| 2 | *G4B84_009351* | 47.11 | 672 | 173 | MFS |
| 3 | *G4B84_005374* | 42.77 | 559 | 59 | MFS |
| 4 | *G4B84_012177* | 41.65 | 546 | 52 | MFS |
| 5 | *AFLA_002854* | 35.88 | 513 | 48 | MFS |
| 6 | *G4B84_001982* | 35.67 | 530 | 65 | MFS |

**Table S2.** **Primers used in this study.**

| **Primers** | **Primer sequences (5^’^-3^’^)** | **Length (bp)** |
| --- | --- | --- |
| **Mutant strain construction** | |  |
| 1982 upF | aattcggatcttccagagatGGATGGAGTCGTGGAGCCTC | 1000 |
| 1982 upR | gcatgcaagcGGTGAATGTAGATGTAGGCACGG |  |
| 1982 pyrGF | tacattcaccGCTTGCATGCCTGCAGCA | 1641 |
| 1982 pyrGR | cgccggattatacttAAGGTATTGAAAAGGGTCGAAGG |  |
| 1982 downF | accttAAGTATAATCCGGCGTTGGTCA | 1000 |
| 1982 downR | ttcaactgccgttcgacgatGTTCAATGGTGTGCCTCTTCTCG |  |
| 1982 F | GCAACTCATAGACCAGGAGG | 1404 |
| 1982 R | CCACCCCAGATGAAGAACAC |  |
| 1982 uupF | ATACGACGAGAAACGGGCAA | 3115 (Δ)  6623 (RT) |
| 1982 pyrGR | cgccggattatacttAAGGTATTGAAAAGGGTCGAAGG |  |
| 1982 pyrGF | tacattcaccGCTTGCATGCCTGCAGCA |  |
| 1982 ddownR | AGTCGAGTCCTGCACACAAG |  |
| 374 upF | aattcggatcttccagagatGAACCAGAAAACTTTACTGTGTGTCTT | 1000 |
| 5374 upR | ggcatgcaagcAATGATGGATGGATTCCTCAATG |  |
| 5374 pyrGF | tccatcattGCTTGCATGCCTGCAGCA | 1641 |
| 5374 pyrGR | ccatAAGGTATTGAAAAGGGTCGAAGG |  |
| 5374 downF | cccttttcaataccttATGGTATCATGTCTAATGAACCAGCT | 1000 |
| 5374 downR | ttcaactgccgttcgacgatCCGGAGAAGCCGATCGTC |  |
| 5374 F | GCCCCGTCAAGTATCTGACA | 1344 |
| 5374 R | GCAAGACCAATGCAGACAGC |  |
| 5374 uupF | CACACCGAGTCACAAACGGA | 2985 (Δ)  6745 (RT) |
| 5374 pyrG R | ccatAAGGTATTGAAAAGGGTCGAAGG |  |
| 5374 pyrG F | tccatcattGCTTGCATGCCTGCAGCA | 2741 |
| 5374 ddownR | CGTCTCCTGGACGGTGTTAC |  |
| 9351 upF | aattcggatcttccagagatACCTTGATGTGATAATGATATAGCCAA | 1000 |
| 9351 upR | ggcatgcaagcTGTCTAGTTTGTAAAAATATATTAAAAATAATGG |  |
| 9351 pyrGF | aactagacaGCTTGCATGCCTGCAGCA | 1641 |
| 9351 pyrGR | tAAGGTATTGAAAAGGGTCGAAGG |  |
| 9351 downF | cgacccttttcaataccttATTGTCATTCCATATTGGTTGTTCA | 1000 |
| 9351 downR | ttcaactgccgttcgacgatTAAATTATAATTTCCAAAATTAGGTTATTATCG |  |
| 9351 F | GGCATTCTTCACGCCGTTCA | 1677 |
| 9351 R | CTCGTTTGCGTTTGCTCTGC |  |
| 9351 uupF | ACAACTATTCCAGCGAACCCT | 3065 |
| 9351 pyrG R | tAAGGTATTGAAAAGGGTCGAAGG |  |
| 9351 pyrG F | aactagacaGCTTGCATGCCTGCAGCA | 3070 |
| 9351 ddownR | TCCGATTCGAACAAGCCTGC |  |
| **Revertant strain construction** | |  |
| 1982 RT1F | aattcggatcttccagagatAAGTATAATCCGGCGTTGGTCA | 2877 |
| 1982 RT1R | ccatCTAGGCCTTCTCAACATGCTCC |  |
| 1982 RT2F | tgttgagaaggcctagATGGGGTGACGATGAGCCG | 2008 |
| 1982 RT2R | catgcaagcGGGCAATTGATTACGGGATCC |  |
| 1982 RT3F | atcaattgcccGCTTGCATGCCTGCAGCA | 1641 |
| 1982 RT3R | ttcaactgccgttcgacgatAAGGTATTGAAAAGGGTCGAAGG |  |
| 1982 uupF | ATACGACGAGAAACGGGCAA | 5138 |
| 1982 PT R | catgcaagcGGGCAATTGATTACGGGATCC |  |
| 1982 PT F | tgttgagaaggcctagATGGGGTGACGATGAGCCG | 4849 |
| 1982 ddownR | AGTCGAGTCCTGCACACAAG |  |
| 5374 RT1F | aattcggatcttccagagatGAACCAGAAAACTTTACTGTGTGTCTT | 2946 |
| 5374 RT1R | tcaccccatTCACATCGGTATATTCAGAGCAAAC |  |
| 5374 RT2F | taccgatgtgaATGGGGTGACGATGAGCCG | 2008 |
| 5374 RT2R | catgcaagcGGGCAATTGATTACGGGATCC |  |
| 5374 RT3F | atcaattgcccGCTTGCATGCCTGCAGCA | 1641 |
| 5374 RT3R | ttcaactgccgttcgacgatAAGGTATTGAAAAGGGTCGAAGG |  |
| 5374 uupF | CACACCGAGTCACAAACGGA | 5248 |
| 5374 PT R | catgcaagcGGGCAATTGATTACGGGATCC |  |
| 5374 PT F | taccgatgtgaATGGGGTGACGATGAGCCG | 4809 |
| 5374 ddownR | CGTCTCCTGGACGGTGTTAC |  |
| **Yeast heterologous expression vector construction** | | |
| 1982F (*BamH*Ⅰ) | tctggtaccacgcgtggatccATGGGCTTCATGTTGAAGAAGC | 1590 |
| 1982R (*BamH*Ⅰ) | tcctttactcccgggggatccGGCCTTCTCAACATGCTCCA |  |
| 5374F (*BamH*Ⅰ) | tctggtaccacgcgtggatccATGGGTGTCTCTAATCTGATGGCC | 1677 |
| 5374R (*BamH*Ⅰ) | tcctttactcccgggggatccCTCGCGGATCTCGGTGGC |  |
| EGFP-F | CTACCTGTTCCATGGCCAAC | 385 |
| EGFP-R | GGTCTGCTAGTTGAACGCTTC |  |
| M13-F (pRS424-*1982*-EGFP) | GTAAAACGACGGCCAGT | 3268 |
| M13-R | CAGGAAACAGCTATGAC |  |
| M13-F (pRS424-*5374*-EGFP) | GTAAAACGACGGCCAGT | 3355 |
| M13-R | CAGGAAACAGCTATGAC |  |
